# Coping strategies used by nurses during the COVID-19 pandemic: A narrative literature review

**DOI:** 10.4102/hsag.v26i0.1652

**Published:** 2021-09-28

**Authors:** Leepile A. Sehularo, Boitumelo J. Molato, Isaac O. Mokgaola, Gopolang Gause

**Affiliations:** 1 NuMIQ Focus Area, School of Nursing Science, Faculty of Health Sciences, North-West University, Mmabatho, South Africa

**Keywords:** coping, coping strategies, nurses, nursing, COVID-19

## Abstract

**Background:**

During the coronavirus disease 2019 (COVID-19) pandemic, it is understandable that nurses are working under stressful conditions. A successful use of effective coping strategies during the COVID-19 pandemic will help nurses to manage stressful conditions.

**Aim:**

The objective of this narrative literature review was to explore and describe the coping strategies used by nurses during the COVID-19 pandemic.

**Setting:**

This study was conducted from all available literature related to the coping strategies used by nurses during the COVID-19 pandemic globally.

**Methods:**

A narrative literature review was conducted to answer researchers’ concern of coping strategies used by nurses during COVID-19. The purposive sampling technique was used to select three online databases that were used to search for the relevant literature, namely Google Scholar, Science Direct and African Journals (formerly SAePublications). Search terms used to conduct this study include coping, coping strategies, nurses, nursing and COVID-19 pandemic. This study included English studies focusing on coping strategies used by nurses during COVID-19 published between 2019 and 2021. The study excluded newspaper articles, conference reports and other databases not mentioned in this study.

**Results:**

The findings identified the following strategies: use of COVID-19 protective measures, avoidance strategy, social support, faith-based practices, psychological support and management support are used by nurses as coping strategies during the COVID-19 pandemic.

**Conclusion:**

The use of the identified coping strategies by nurses may reduce stress and burnout during the COVID-19 pandemic. Recommendations were made for future research, nursing education and practice.

**Contribution:**

This is the first narrative literature review focusing on the coping strategies used by nurses during the COVID-19 pandemic. The findings of this narrative literature review provide insight that may be used by nurses of all categories to cope during the COVID-19 pandemic.

## Introduction

The world has experienced several pandemics of contagious diseases in the past two decades such as Middle East Respiratory Syndrome (MERS), Ebola and Zika from 2014 to 2016, H1N1 in 2009 and SARS in 2003 (Zhang et al. 2020:2). The novel coronavirus Severe Acute Respiratory Syndrome-Coronavirus 2 (SARS-CoV-2), which causes the disease COVID-19, was first identified in Wuhan, China in December 2019 and declared as a worldwide pandemic by the World Health Organisation (WHO) on the 11th of March 2020 (Huffman et al. 2020:1; Man et al. 2020:1). There are over 14.2 million COVID-19 cases and 600 000 deaths worldwide and rising as of 19th July 2020 (Mellins et al. 2020:62). By early May 2020, more than 90 000 healthcare workers (HCWs) were diagnosed with COVID-19 in 30 countries, with 260 deaths of nurses (Dramowski et al. 2020:2). In South Africa (SA), on 6 May 2020, Minister of Health Dr Zweli Mkhize reported that 511 HCWs had tested positive for SARS-CoV-2 (7% of the national total), with nurses accounting for 53% of total HCW cases (Dramowski et al. 2020:2). Based on these statistics, authors such as Rees et al. (2020:309) argued that protecting HCWs from COVID-19 is a global priority. The nursing profession, the most trusted and respected, has been on the front lines, racing to care for the emotional and physical needs of their patients and families whilst struggling to keep their own worries for self and loved ones at bay. In times of crisis such as COVID-19 pandemic, nurses have been there to bring care and comfort to those in need (Ward-Miller et al. 2020:1).

Nurses work directly with COVID-19 suspected or confirmed patients and suffer from enormous psychological pressure even if taking precautions in advance (Cui et al. 2021:586). Healthcare workers such as nurses are more likely to be infected by COVID-19 than any other group (Cui et al. 2021:586). Nurses are experiencing emotional and mental stress because of COVID-19. On the other hand, authors such as Ali, Astin Cole and Sa’d Hamasha (2020:2064) mentioned that nurses experience work-related stress, resulting from taking care of patients infected with conditions such as COVID-19 and stress caused by receiving more patients. Other nurses are also worried about transmitting COVID-19 to their family, friends and colleagues (Ali et al. 2020:2064; Cui et al. 2021:591; Man et al. 2020:2; Shaohua et al. 2020:11; Shechter et al. 2020:5). How nurses cope with the given challenges remains largely unknown. According to Lazarus and Folkman’s Transactional Model to Stress and Coping (Dardas & Ahmad 2015:^5^), coping refers to a cognitive and behavioural efforts that are constantly changing to master, reduce or tolerate a specific stressor appraised as exceeding one’s available resources and abilities (Dardas & Ahmad 2015:^5^). The same model refers to coping strategies as the intermediate process between stressors and health outcomes (Dardas & Ahmad 2015:^5^). Coping strategies are usually individualised and influenced by personal experiences, education levels and resources available for them in a social context (Zhao et al. 2021:888). Literature suggests that because of the fear of getting their family and friends infected by COVID-19, nurses may not be spending much time engaging with family and friends (Ali et al. 2020:2065). A study conducted in New York by Shechter et al. (2020:5) added that most nurses are highly distressed by having to maintain ‘social distance’ from family. These are the people who should be assisting the nurses to cope during the COVID-19 pandemic. This creates a gap in social support that can be addressed through compassionate management policies. Gunawan et al. (2021) add that roles and responsibilities of nurses are crucial in the battle of COVID-19, but nursing duties also put them at risk of infections such as COVID-19. According to Cui et al. (2021:585), nurses face higher risks of death than medical doctors in some countries. This is why some of them are unable to deal with the stress of working with COVID-19 patients.

It is clear from the given information that COVID-19 has not only had an impact on nurses’ emotions, but their coping strategies too have undergone a change (Huang et al. 2020:2). Coronavirus disease of 2019 challenges cause nurses to worry more about their friends and family members, subsequently making them more stressed, anxious and more inclined to adopt negative coping strategies (Cui et al. 2021:591). Hence, authors such as Zhang et al. (2020:1) and Kar, Kar and Kar (2021:3) mentioned that coping strategies are needed to reduce their stress and burnout. The given information shows that there are many gaps that should be addressed on the issue of COVID-19. Some of these gaps include coping strategies used by nurses during the COVID-19 pandemic.

Whilst studies have investigated nurses’ stress levels during pandemics, there are still gaps in the discussion regarding how the nurses cope (Ali et al. 2020:2059). Based on the given discussion, the following review question was asked:

- What are the coping strategies used by nurses during the COVID-19 pandemic?

## Aim

This study aimed to explore and describe the coping strategies used by nurses during the COVID-19 pandemic reported in the literature.

## Design and methods

**FIGURE 1:**
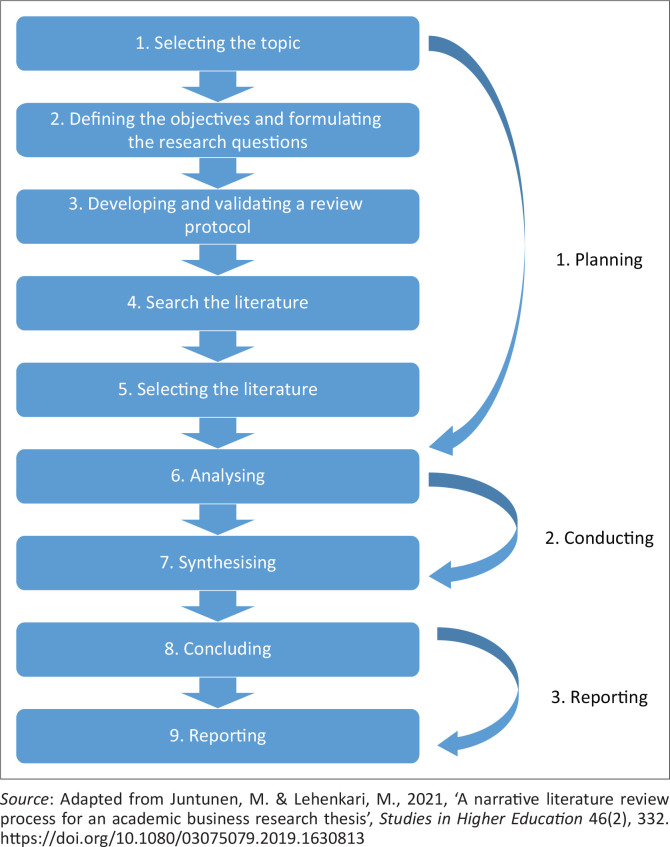
Phases and steps of a narrative literature review.

A narrative literature review was conducted to address the concern on the coping strategies used by nurses during the COVID-19 pandemic. Literature review involves finding, reading, understanding and forming conclusions about the published research and theory and presenting it in an organised manner (Brink, Van der Walt & Van Rensburg 2012:71). The purpose of using a narrative literature review in this study was to provide an up-to-date account of what is already known about the coping strategies used by nurses during the COVID-19 pandemic. The three phases and nine steps of a narrative literature review were adopted to answer the review question (Juntunen & Lehenkari 2021:332). The phases include planning, conducting and reporting. Figure 1 depicts the phases and steps of a narrative literature review followed in this study:

Step 1: Selecting the topic – The topic selected for this study is coping strategies used by nurses during the COVID-19 pandemic.

Step 2: Defining the objectives and formulating the research questions – The objective of this study was to explore and describe the coping strategies used by nurses during the COVID-19 pandemic. This study aimed to address the following review question:

- What are the coping strategies used by nurses during the COVID-19 pandemic?

Step 3: Developing and validating a review protocol – This step is comparable to research design in empirical research, and it contains a pre-set plan for how researchers aimed to conduct all other steps of the research process. In this context, the first author conceptualised the study. Validation of the protocol was performed by the second, third and fourth authors. All authors contributed equally in finalising the manuscript, and they all agreed that it is ready for submission to an accredited journal such as *Health South Africa Gesondheid* (HSAG).

Step 4: Search the literature – Purposive sampling technique was used to select the three online databases used to search for the relevant literature, namely Google Scholar, Science Direct and African Journals (formerly SAePublications). Search terms used to conduct this study include coping, coping strategies, nurses, nursing and COVID-19 pandemic. Table 1 shows how literature was searched.

**TABLE 1:** Literature search.

Databases	Search terms	Inclusion criteria	Exclusion criteria
Google Scholar Science Direct African Journals (formerly SAePublications)	Coping Coping strategies Nurses Nursing COVID-19 pandemic	English studies focusing on coping strategies used by nurses during COVID-19 published between 2019 and 2021	Newspaper articles Conference reports Other databases not mentioned in this study

Step 5: Selecting the literature – This step refers to deciding which articles are included in the analysis or excluded from the analysis (Juntunen & Lehenkari 2021:333). All authors agreed on the inclusion and exclusion criteria of this study. This study included English studies focusing on coping strategies used by nurses during COVID-19 published between 2019 and 2021. The reason for including English studies is that all authors are conversant with English language including the majority of the readers of HSAG. The reason for including studies published between 2019 and 2021 is COVID-19 started in 2019, and the majority of authors have been publishing since that time until 2021 when the manuscript was written. The study excluded newspaper articles, conference reports and other databases not mentioned in this study.

Step 6: Analysing – This step includes reading and re-reading selected articles and making sense of them. The step also includes coding concepts and themes so that similar data are categorised and grouped together. All authors of this manuscript contributed in reading and re-reading selected articles and grouped similar data together. The reason for all authors to participate in this step was to minimise errors in the whole review especially data extraction process. Johns Hopkins Nursing Evidence-Based Practice Research Evidence Appraisal tool as explained by Newhouse et al. (2007) was used by all authors to grade evidence in this study. Quality guides of the tool include High Quality, which is written as A, Good Quality which is written as B and Low Quality or Major Flaws, which is written as C. Quality of the studies was based on the author(s), year, aim, design, sample and results.

Step 7: Synthesising – This step refers to organising the grouped data into a specific structure (Juntunen & Lehenkari 2021:333). All authors of this manuscript separately grouped similar data together and met for finalising the themes. Six themes emerged from the findings of the study.

Step 8: Concluding – In line with Juntunen and Lehenkari (2021:334), this step includes the demonstration of how the findings of this study extend the existing research, the implications for research, academics and nurse practitioners. Recommendations were made for future research, nursing education and practice. This step also includes the methodological limitations of the review.

Step 9: Reporting – In line with Juntunen and Lehenkari (2021:334), the structure of this literature review includes the title, abstract, introduction, design and methods, discussion, conclusion and list of references.

### Ethical considerations

Researchers conducted this study competently, rigorously and methodologically sound (Brink et al. 2012:32). They conducted this study ethically from the conceptualisation, planning, implementation and dissemination phases. All authors contributed differently in writing this manuscript as indicated in the methodology section. Authors with different experience in writing reviews increased trustworthiness of the study. No permission was needed to conduct this study. All sources used in this study have been duly acknowledged in text and at the reference list.

## Results

### Themes in literature

**FIGURE 2:**
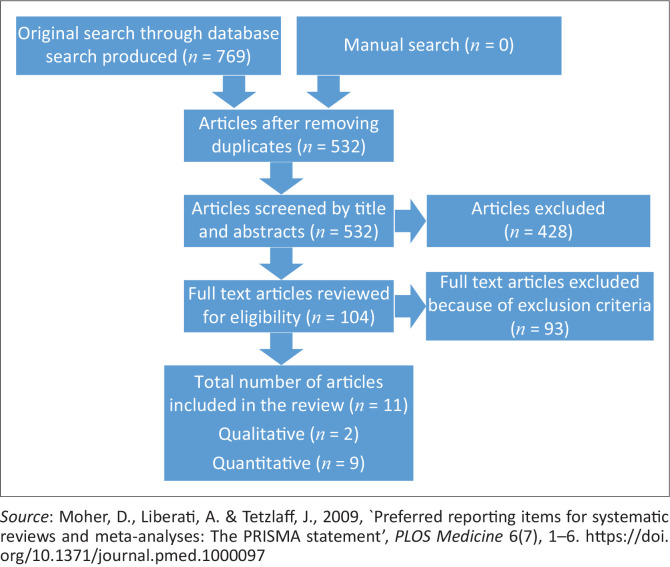
Flow chart of the search strategy.

The findings from the search are given in Figure 2. Six themes emerged from the findings of this study (see Table 2 for reviewed studies), namely use of COVID-19 protective measures, avoidance strategy, social support, faith-based practices, psychological support and management support.

**TABLE 2:** Description of reviewed studies (A = High Quality, B = Good Quality, C = Low Quality of Major Flaws).

Author(s) and year	Aim	Design and sample	Rigor
Ali et al. 2020	The study aimed to investigate the major stressors and coping strategies reported by nurses working directly with potentially infectious patients in Alabama, United States, during the COVID-19 pandemic.	Cross-sectional survey, 109 nurses.	Aim and objectives clear Design relevant Results consistent Implications discussed **Quality Appraisal= High Quality (A)**
Cai et al. 2020	The study aimed to investigate psychological impact and coping strategies of frontline medical staff in Hunan Province during the COVID-19 outbreak between January and March 2020.	Cross-sectional observational study, 534 frontline medical staff.	Aim and objectives clear Design relevant Results consistent Implications discussed **Quality Appraisal= High Quality (A)**
Cui et al. 2021	To identify the impact of COVID-19 on the pathology of Chinese nurses in emergency departments and fever clinics and to identify associated factors.	Online cross-sectional study, 453 emergency nurses.	Aim and objectives clear Design relevant Results consistent Implications discussed **Quality Appraisal= High Quality (A)**
Gunawan et al. 2021	The aim of the study was to explore the lived experience of nurses in combating COVID-19 in Belitung, Indonesia.	Phenomenological design, 17 clinical nurses.	Aim and objectives clear Design relevant Results consistent Implications discussed **Quality Appraisal= High Quality (A)**
Huang et al. 2020	The study aimed to explore the current status and relationship of emotional responses and coping strategies of nurses at all levels of hospitals in Anhui Province.	Online survey, 802 nursing and nursing students.	Aim and objectives clear Design relevant Results consistent Implications discussed **Quality Appraisal= High Quality (A)**
Munawar and Choudhry 2020	The study aimed to examine the psychological impact of COVID-19 on emergency HCWs and to understand how they are dealing with COVID-19 pandemic, their stress-coping strategies or protective factors and challenges whilst dealing with COVID-19 patients.	Qualitative inquiry, 15 frontline emergency HCWs.	Aim and objectives clear Design relevant Results consistent Implications discussed **Quality Appraisal= High Quality (A)**
Savitsky et al. 2020	The study aimed to assess levels of anxiety and ways of coping amongst nursing students in the Ashkelon Academic College, Southern District, Israel.	Cross-sectional study, 244 nursing students.	Aim and objectives clear Design relevant Results consistent Implications discussed **Quality Appraisal= High Quality (A)**
Shahrour and Dardas 2020	The study aimed to establish the prevalence of acute stress disorder and predictors of psychological distress amongst Jordanian nurses.	Quantitative, cross-sectional, descriptive and comparative design, 448 Jordanian nurses.	Aim and objectives clear Design relevant Results consistent Implications discussed **Quality Appraisal= High Quality (A)**
Shaohua et al. 2020	The study aimed to explore the relationship between work stressors and mental health in frontline nurses exposed to COVID-19.	Cross-sectional study, 723 frontline nurses.	Aim and objectives clear Design relevant Results consistent Implications discussed **Quality Appraisal= High Quality (A)**
Sheroun et al. 2020	The aim of the study was to assess the perceived stress and coping strategies amidst COVID-19 lockdown amongst the BSc nursing students studying in nursing colleges located in Pune.	Cross-sectional descriptive study, 427 nursing students.	Aim and objectives clear Design relevant Results consistent Implications discussed **Quality Appraisal=High Quality (A)**
Zhang et al. 2020	The aim of the study was to identify stressors and burnout amongst frontline nurses caring for COVID-19 patients in Wuhan and Shanghai and to explore perceived effective morale support strategies.	Cross-sectional survey, 110 nurses.	Aim and objectives clear Design relevant Results consistent Implications discussed **Quality Appraisal= High Quality (A)**

*Source*: Adapted from Kangasniemi, M., Pakkanen, P. & Korhonen, A. 2015, ‘Professional ethics in nursing: an integrative review’, *Journal of Advanced Nursing,* 71(8), 1744–1757. HCW, healthcare worker.

### Use of COVID-19 protective measures

The first theme identified from the literature as a strategy used by nurses during the COVID-19 pandemic was the use of COVID-19 protective measures. Other synonyms for protective measures include preventive or precautionary measures. In this study, the word ‘protective measures’ will be used consistently. The aim of these measures is to slow the transmission of COVID-19. Therefore, public compliance with these measures is important in successful control of COVID-19 pandemic (Padidar et al. 2021:1). Seven articles addressed the use of COVID-19 protective measures (Ali et al. 2020; Cai et al. 2020; Cui et al. 2021; Huang et al. 2020; Sheroun et al. 2020; Shahrour and Dardas 2020; Zhang et al. 2020) A cross-sectional online study conducted by Sheroun et al. (2020:287) on the nursing students found that COVID-19 protective measures have been used by the masses in different ways, along with the mentioned coping styles avoiding public places or events, washing or disinfecting hands more often than usual, avoiding public transports such as buses and trains. Awareness of these protective measures with reduced numbers of reported cases reduce the stress of the medical staff such as the nurses (Cai et al. 2020:14). A study by Shahrour and Dardas (2020:1692) indicated that nurse managers need to take an active role in ensuring the personal safety of their staff through working closely with the hospital management in securing and providing personal safety measures. Huang et al. (2020:10) added that hospitals arrange adequate medical protective equipment and develop a broad range of interventions to block the spread of infectious diseases such as COVID-19 so as to form a safe environment where COVID-19 stops spreading in hospitals. This creates an optimistic environment and guarantees the personal safety of nurses, thereby enabling them to carry on with the highest quality of patient care to win the battle against the COVID-19 pandemic. In contrast, a study by Ali et al. (2020:2065) mentioned that protective measures are not readily used by all nurses as coping strategies. The study indicated that only 75% of the participants reported that they follow all strict protective measures such as protective gear, face masks and hand washing to reduce their risk of infection. Cui et al. (2021:591) added that nurses should be trained for the skills necessary to protect themselves from COVID-19. Adequate understanding of COVID-19 could increase nurses’ confidence and sufficient training should be offered (Zhang et al. 2020:7).

### Avoidance strategy

The second theme identified from the literature as a strategy used by nurses during the COVID-19 pandemic is avoidance strategy. Avoidance in this study refers to the act or practice of withdrawing or avoiding something unwanted. Only two articles indicated avoidance strategy as a strategy used by nurses during the COVID-19 pandemic (Ali et al. 2020; Sheroun et al. 2020). A study conducted in Alabama by Ali et al. (2020:2065) found that majority of the participants reported avoiding media coverage providing updates on COVID-19 infection and mortality statistics. According to Sheroun et al. (2020:286), erroneous news reports have also added to anxiety and fear. However, a study by Ali et al. (2020:2065) pointed that a reliance on avoidance strategy for nurses could significantly limit their access to updated risk information, which may include improved studies or additional protective measures.

### Social support

The third theme identified from literature as a strategy used by nurses during the COVID-19 pandemic is social support. Social support is one of the most effective means by which people can cope with stressful events, and it may come from spouse, relatives, friends, co-workers and community (Kim et al. 2008:518) Previous research on social support considered it as functional strategy to cope with problematic situations (Babore et al. 2020:4). Five articles that mentioned social support as a strategy used by nurses during the COVID-19 pandemic are Gunawan et al. (2021), Shaohua et al. (2020), Zhang et al. (2020), Cai et al. (2020) and Ali et al. (2020). Nurses should be supported at all the times to make them happy at work (Gunawan et al. 2021). With the help of social support, nurses’ stress responses can be significantly reduced (Shaohua et al. 2020:3). For instance, family support is highly valued by the nurses during these stressful periods of COVID-19 (Cai et al. 2020:14; Zhang et al. 2020:5). More specifically, nurses are encouraged to speak with a loved one through a video chat or receive individual therapy at least once during their shift (Ali et al. 2020:2065).

### Faith-based practices

The fourth theme identified from literature as a strategy used by nurses during the COVID-19 pandemic is faith-based practices. Only two from the nine articles used in this literature review mentioned faith-based practices as a strategy used by nurses in COVID-19 pandemic (Munawar & Choudhry 2020; Savitsky et al. 2020). The faith-based practices and belief system are seen to play an integral role in the lives of the health workers such as nurses to cope with the COVID-19 pandemic (Munawar & Choudhry 2020:4). One of the participants in a qualitative study conducted in Pakistan by Munawar and Choudhry (2020:4) said ‘My coping is based on my faith that every illness, disease or virus comes from God and it cannot harm us without His will, so COVID-19 is no exception’ (Munawar & Choudhry 2020:4). Coping is considered to be of critical importance in determining whether a stressful event results in adaptive or maladaptive outcomes (Dardas & Ahmad 2015:^5^). In contrast, a study by Savitsky et al. (2020:5) found that anxiety level of religious student nurses might increase because during COVID-19 period living a religious lifestyle was seriously compromised because of mandatory prohibitions against praying in mosque or synagogue and using a Mikveh (Jewish ritual bath). The given information highlights the gap that needs to be addressed on how to assist nurses to continue with their religious lifestyle during the COVID-19 pandemic.

### Psychological support

The fifth theme identified from literature as a strategy used by nurses during the COVID-19 pandemic is a psychological support. Psychological support helps to relieve emotional suffering so that beneficiaries are sooner able to rely on their own resources and cope more successfully with the hardships they face on the road to recovery (International Federation of Red Cross and Red Crescent Societies 2003:24). According to Cole et al. (2020:2), psychological support should aim at addressing the mental health challenges that have emerged because of trauma or other distressing experiences on the frontline. Only four articles mentioned that the nurses use psychological support as a strategy to cope with the COVID-19 pandemic (Huang et al. 2020; Sheroun et al. 2020; Zhang et al. 2020). A study conducted in Anhui Province in China by Huang et al. (2020:1) found that hospitals focus on providing psychological support to the nurses and training in coping strategies. A study by Zhang et al. (2020:6) found that continuous attention and psychological support must be applied in a timely manner. These authors add that psychological support should be provided by management and organisations that meet the needs of the nurses. Sheroun et al. (2020:287) added that providing nursing students with psychological support and assurance help those nursing students to overcome the stress to cope in the lockdown and perform better in their studies. Management should also timely identify the crux of psychological problems of the nurses and attend to them. Hospital managers should also focus on stimulating nurses’ potentially positive traits and encourage them to adopt effective coping strategies and social support to reduce adverse psychological levels (Shaohua et al. 2020:13). This shows another gap that more studies should be conducted on how all nursing categories are coping during the COVID-19 pandemic.

### Managerial support

The sixth last theme identified from the literature as a strategy used by nurses during the COVID-19 pandemic is the managerial support. Managers have a critical role to play in supporting the needs of their employees. Only three themes address managerial support as a strategy used by nurses during the COVID-19 pandemic (Ali et al. 2020; Shahrour & Dardas 2020; Shaohua et al. 2020). A study by Shaohua et al. (2020:11) indicated that management should play great attention to the work pressure and mental states of the nurses whilst fighting COVID-19. Management should also timely identify the crux of psychological problems of the nurses and attend to them. Hospital managers should also focus on stimulating nurses’ potentially positive traits and encouraging them to adopt effective coping strategies and social support to reduce adverse psychological levels (Shaohua et al. 2020:13). A study by Ali et al. (2020:2065) found that the importance of hospital administration provided psychological support as a crucial factor in the nurses’ ability to overcome their challenges caused by COVID-19. Nurse managers can take a leading role in implementing stress-reduction strategies for nurses through providing consecutive rest days, rotating allocations of complex patients, arranging support services and being accessible to the nursing staff (Shahrour & Dardas 2020:1692).

## Discussion

This study aimed to explore and describe the coping strategies used by nurses during the COVID-19 pandemic. To the knowledge of the authors, this is the first study to be conducted on this topic. A literature review design and methods were used to address the researchers’ concern of coping strategies used by nurses during COVID-19. Eleven articles were found to be relevant to address the researchers’ concern of coping strategies used by nurses during COVID-19 (Ali et al. 2020; Cai et al. 2020; Cui et al. 2021; Gunawan et al. 2021; Huang et al. 2020; Munawar & Choudhry 2020; Savitsky et al. 2020; Shahrour & Dardas 2020; Shaohua et al. 2020; Sheroun et al. 2020; Zhang et al. 2020). The findings revealed that COVID-19 protective measures, avoidance strategy, social support, faith-based practices, psychological support and management support are used by nurses as coping strategies during the COVID-19 pandemic. These findings are consistent with a study by Htay et al. (2021:5) who found that health professionals coped with their distress during the COVID-19 pandemic using a plethora of techniques ranging from psychological, social and religious or spiritual approaches. Almost all studies mentioned COVID-19 protective measures as a strategy used by the nurses during COVID-19. All countries affected by the COVID-19 pandemic use strategies such as social distancing, hand washing and wearing of masks to prevent and cope with COVID-19. This was also supported with hard lockdowns. This study revealed that social support is effective in coping with stress during the COVID-19 pandemic. This finding is supported by Cai et al. (2020:14) who indicated that during pandemics such as COVID-19, support from family and friends and positive attitude have previously been shown to reduce stress. According to Htay et al. (2021:2), the World Health Organisation (WHO) advised the healthcare workers such as nurses for self-care particularly for maintaining healthy lifestyles and getting informal social support. Faith-based practices are also used by nurses to cope with COVID-19. However, in some countries such as South Africa, lockdowns made faith-based practices for majority of the people including the nurses to be impossible. This finding is supported by Htay et al. (2021:6) who found that faith in the benevolent God may promote positive thinking and hope. Psychological support has been mentioned in three studies to be used by nurses during COVID-19 (Huang et al. 2020:1; Sheroun et al. 2020:287; Zhang et al. 2020:6). A study by Chirico, Nucera and Magnavita (2020:2) added that psychological intervention should include two pillars. Firstly, it should provide healthcare workers such as nurses with adequate information, training and PPE in order to tackle and cope with COVID-19. Secondly, it should enhance emotional skills of healthcare workers to cope with anxiety. Managerial support was the last theme identified from the findings of this study as a coping strategy used by nurses during COVID-19. A study by Zhao et al. (2021:892) added that effective coping strategies included approachability of management, peer support and teamwork. The information obtained from the findings of this study may assist nurses to cope effectively with the challenges caused by the COVID-19 pandemic. These findings should inform the development and implementation of effective interventions for improving the nurses’ coping strategies during the COVID-19 pandemic.

### Limitations

This study has a number of limitations. Firstly, the focus of this literature review was on the nurses only. Therefore, findings of this study cannot be generalised to other professionals. Secondly, the study focused on coping strategies used during the COVID-19 pandemic only, coping strategies used by nurses during other pandemics such as MERS, Ebola, Zika, H1N1 and SARS might be relevant during COVID-19. Thirdly, there were very few articles that met the inclusion criteria of this study. This shows that more studies are needed on the topic of COVID-19 particularly amongst the nurses. Fourthly, studies written in other languages other than English might have provided more insight into the coping strategies used by nurses during the COVID-19 pandemic. Lastly, there is a need for a study focusing on effectiveness of nurses coping strategies during the COVID-19 pandemic. The given limitations might be addressed by future studies on this topic.

### Recommendations

There is a need for more studies on the coping strategies used by nurses during the COVID-19 pandemic. These studies should follow different methodologies such as quantitative, qualitative and mixed methods. There is also a need to examine the effects of different coping strategies on stress levels or other outcomes. Nurses at the hospitals and clinics should be educated on the effective strategies to use in order to cope with stress and burnout during COVID-19. Government and management from hospitals and clinics should support nurses to cope with stress during COVID-19.

## Conclusion

The aim of this literature review was to explore and describe the coping strategies used by nurses during the COVID-19 pandemic. This is the first study to be conducted on this topic. Six themes emerged from the findings of this study namely, COVID-19 protective measures, avoidance strategy, social support, faith-based practices, psychological support and management support. These findings show that the use of effective coping strategies by nurses may reduce stress during the COVID-19 pandemic. Recommendations were made for future studies, nursing education and practice.
